# Recent progress on single-molecule localization microscopy

**DOI:** 10.52601/bpr.2021.210023

**Published:** 2021-10-31

**Authors:** Lusheng Gu, Wei Ji

**Affiliations:** 1 Institute of Biophysics, Chinese Academy of Sciences, Beijing 100101, China; 2 College of Life Science, University of Chinese Academy of Sciences, Beijing 100049, China; 3 Bioland Laboratory (Guangzhou Regenerative Medicine and Health Guangdong Laboratory), Guangzhou 510320, China

**Keywords:** Single-molecule localization microscopy, Super-resolution imaging, Centroid fitting, Localization precision

## Abstract

Super-resolution imaging based on single-molecule localization has been developed for more than a decade. These techniques can break through diffraction limit of fluorescent microscopy and initially improve the resolution by an order of magnitude to ~20 nm, by introducing photoactivatable/photoswitching probes and centroid fitting method. As the demand of biological research, the localization precision of single-molecules was further improved by several state-of-the-art methods in the past several years. This review focuses on the latest developed techniques which have greatly improved the performance of single-molecule localization microscopy, from measurement principle to hardware design. These methods are essential for the study of nanostructures and biomacromolecule dynamics inside of cells.

## INTRODUCTION

Fluorescent microscopy is a powerful tool for biological and biomedical research to investigate structures of organelle, intracellular activities, and biomolecule functions because of its noninvasiveness and high specificity (BETZIG* et al.* 1991; Denk* et al.* 1990; Patterson* et al.* 1997). However, the resolution was limited to ~200 nm in the lateral direction and ~500 nm in the axial direction due to diffraction of light (Axelrod 2001; Lichtman and Conchello 2005; Semwogerere and Weeks 2005). Two point sources that are closer than the half wavelength of light could not be resolved by a light microscope. As a consequence, the nanostructures in cells hindered behind this limit could not be directly resolved by fluorescent microscopy (BETZIG* et al.* 1991; Stephens and Allan 2003).

Three types of techniques have been developed recently to break through the resolution limit of fluorescent microscopy as list below, which are widely used in biology research.

(1) Structured illumination microscope (SIM), which illuminated the sample with a structured pattern, thus shifts the high frequency signals into the low frequency domain which could be acquired by the microscope (Gustafsson 2000; Saxena* et al.* 2015). Then the high frequency information will be restored and shifted to the corresponding frequency domain, and the image with higher resolution could be reconstructed from several images. This method was extended to higher order with nonlinear schemes, and 3D SIM was also developed to resolve the 3D structure in cells (Guo* et al.* 2018; Gustafsson 2005; Huang* et al.* 2018; Li* et al.* 2015; Schermelleh* et al.* 2008).

(2) The stimulated depletion microscope (STED) is another approach to break the diffraction limit (Hell 2003; Hell and Wichmann 1994; Klar and Hell 1999; Klar* et al.* 2000). The excited fluorescent molecule is also illuminated by the depletion light, which will transfer the excited molecules into the ground state. The depletion light is modulated into a doughnut shape so that molecules near the center are less possible to be depleted. So, only the molecules near the center of the doughnut are kept in an emission state and the fluorescence signal from them is collected. With this method, the effective size of PSF could be decreased with the increasing intensity of the depletion light, resulting in resolution improvement (Blom and Widengren 2017; Eggeling* et al.* 2009; Hell 2007; Westphal* et al.* 2008).

(3) The single-molecule localization microscopy (SMLM) represents a series of superresolution imaging techniques based on single-molecule localization which have been developed since 2006, such as fluorescent photoactivated localization microscopy ((f)PALM) (Betzig* et al.* 2006; Hess* et al.* 2006), stochastic optical reconstruction microscopy (STORM) (Rust* et al.* 2006), direct STORM (dSTORM) (Endesfelder and Heilemann 2015; Van de Linde* et al.* 2011), binding activation localization microscopy (BALM) (Ries* et al.* 2013; Schoen* et al.* 2011; Szczurek* et al.* 2017), point accumulation for imaging in nanoscale topography (PAINT) (Dai* et al.* 2016; Jungmann* et al.* 2014). The fundamental of these methods was based on the precise localization of isolated molecules. Once an isolated molecule was imaged, the molecule position could be estimated more precisely than the diffraction limit size (Ober* et al.* 2004; Thompson* et al.* 2002). Thus, when enough single-molecule data are accumulated, the interested nanostructure could be resolved with much higher resolution than conventional imaging, and the resolution is mainly depending on the single-molecule localization precision. This technique has been used to study the ultrastructures in cells such as cytoskeleton (Pan* et al.* 2018, 2019), membrane (Shim* et al.* 2012), and even protein–protein interactions in recent years (Liu* et al.* 2014; Wang* et al.* 2017; Yan* et al.* 2019; Zhou* et al.* 2020a). In this review, we focus on the development of SMLM techniques, which yields the highest resolution ability among the super-resolution methods.

## SINGLE-MOLECULE LOCALIZATION MICROSCOPY

### Single-molecule localization

When the light from a point source passes through an imaging system, the image will be an enlarged spot because of the diffraction nature of the light. This spot is called point spread function (PSF) (Lichtman and Conchello 2005; Semwogerere and Weeks 2005). Usually, the full width at half maximum (FWHM) of PSF was used to describe the resolution of the imaging system. When imaging a single-molecule, the nanometer scale molecule could be simplified as a point source, and the image of it could be considered as the PSF of the imaging system. For most imaging systems, Gaussian function approximation can be used to represent the PSF. Then the precision of position (*σ*_loc_) estimation of the single molecule could be *σ*_psf_/$ \sqrt{N} $ (Cheezum* et al.* 2001; Ober* et al.* 2004; Thompson* et al.* 2002), where “*σ*_loc_” is the localization precision, “*σ*_psf_” is the standard derivation (std.) and “*N*” is the photon number collected by the detector. The precision is calculated as std. and the relation between FWHM and std. was $ \mathrm{F}\mathrm{W}\mathrm{H}\mathrm{M}=2\sqrt{2\mathrm{l}\mathrm{o}\mathrm{g}\left(2\right)}\sigma \approx 2.355\sigma $.

A more complicated description of the localization precision which also considers the noise and pixel size effects could be found in the previous report (Thompson* et al.* 2002).

**Figure 1:**
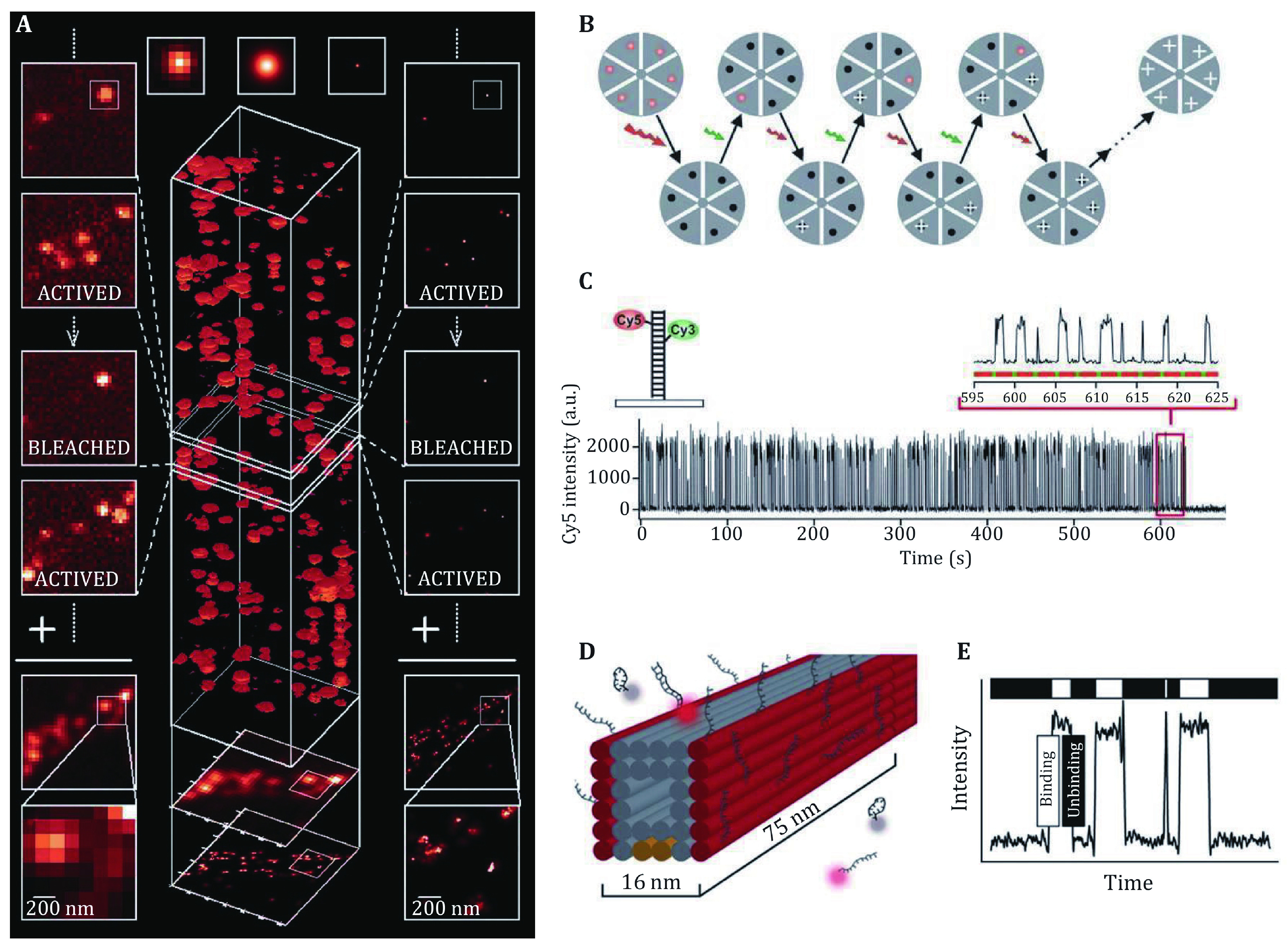
**A** Working principle of PALM with the photoactivatable fluorescent protein. Adapted from Betzig *et al*. (2006) with permission. **B**, **C** Working principle of STORM with photoactivatable Cy3–Cy5 dye pair. **D**, **E** Working principle of DNA-PAINT, the immobilized imager strand could be imaged while the imager strands in solution (**D**) will not causing the fluctuation around the binding sites (**E**)

The SMLM concept was first implemented as photoactivated localization microscopy (PALM) in 2006 (Betzig* et al.* 2006; Hess* et al.* 2006). In this method, the photo-active fluorescent protein (PAFP) was expressed in the cell. When illuminated by a pulse of low intensity 405 nm laser, a small amount of PAFP will be activated and imaged. After all the active PAFPs were turned off, another pulse of 405 nm laser will be applied. This imaging cycle will carry on until enough single-molecule data have been collected, these data could be used to reconstruct super-resolution images of the cellular structure (Fig. 1).

Xiaowei Zhuang’s group proposed a similar method called STORM in 2006 (Rust* et al.* 2006). In the method, they used Cy3-Cy5 dye pair instead of PAFP to label the sample. This dye pair could be switched on by 532 nm laser and excited by 633 nm laser. First, the active dye will be turned off by a high-power 633 nm laser, then a pulse of low intensity of 532 nm laser will be applied to activate a small amount of the dye pair, then the active dye will be excited and imaged by the microscope, until bleached or become inactive. This imaging process will be carried on until enough molecules were collected and a super-resolution image was reconstructed.

Another method to get the single-molecule image was reported based on the binding dynamic characteristics of the single molecule (Sharonov and Hochstrasser 2006). Then this method was developed into another SMLM method called DNA-Point Accumulation for Imaging to Nanoscale Topography (DNA-PAINT) (Dai* et al.* 2016; Jungmann* et al.* 2014; Schnitzbauer* et al.* 2017). In this method, the switching of fluorescent molecules was based on the binding and dissociation dynamics of two complementary single strand DNA. A short DNA strand was used for labeling, and the complementary DNA strand was labeled with the fluorescent dye and called “imager”. When an appropriate concentration of imager exists, it will bind to the target DNA strand and then dissociate spontaneously. When the imager binds to the target DNA strand, the dye on the imager will be immobilized and imaged by the microscope. Otherwise, the imager in the solution will not be imaged because of the thin layer of TIRF illumination and fast dynamics of the imager. This method does not need photoactivatable or photoswitchable probes as in PALM and STORM. Thus, the very bright dye could be used in DNA-PAINT methods such as Atto-655 and Cy3B, which results in several tens of thousands of photons per localization and improvement of localization precision.

The DNA-PAINT method was also extended to multi-color imaging, with a solution exchange strategy called Exchange-PAINT. Multiple imagers will be applied in Exchange-PAINT with the same dye, so there’s no chromatic aberration between each channel. Several works have been reported to reduce the background fluorescence in DNA-PAINT, which will extend this method to a higher imaging depth (Geertsema* et al.* 2021; Schueder* et al.* 2017; Szczurek* et al.* 2017). Another work was reported on the potential of quantitative analysis with DNA-PAINT (Jungmann* et al.* 2016). Also, improving the imaging speed with optimized imaging conditions and specially designed imager strands were reported (Brockman* et al.* 2020; Schueder* et al.* 2019; Strauss and Jungmann 2020).

### Single-molecule analysis

The single-molecule locating algorithm and data analysis algorithm are important aspects of SMLM, and the reconstruction performance relies mainly on the imaging analysis methods (Berglund* et al.* 2008; Cheezum* et al.* 2001; McGorty* et al.* 2014; Ober* et al.* 2004; Quirin* et al.* 2012; Thompson* et al.* 2002). Fitting with 2D Gaussian model is the most widely used localization method, which yields higher precision than the weighted centroid and high fitting speed with GPU acceleration (Henriques* et al.* 2010; Przybylski* et al.* 2017; Quan* et al.* 2010). And, to improve the precision and accuracy with higher aberration introduced by imperfect optics or orientation of molecules, fitting with theoretical PSF or experimental PSF was applied (Smith* et al.* 2010; Zhang* et al.* 2013). Experimentally acquired PSF was also used for fitting, and yields better precision as well as accuracy when fitting single-molecule images (Li* et al.* 2020; Li* et al.* 2018).

To improve the imaging speed, more molecules should be localized within each frame. So the corresponding analysis algorithm to analyze high density molecules was implemented to resolve the partially overlapped molecules (Gu* et al.* 2014; Huang* et al.* 2011; Zhu* et al.* 2012).

The resolution evaluation is another important aspect to judge the imaging condition of the reconstructed image from SMLM. SMLM imaging yields the sparse point cloud that is different from conventional microscopy, which could not be used to calculate the resolution directly. The widely used method is to measure the intensity profile of a fine structure such as the filaments of the microtubule or actin or the membrane, which shows the local resolution of SMLM. There're several methods used to evaluate the global resolution with Fourier analysis or autocorrelation analysis (Descloux* et al.* 2019; Nieuwenhuizen* et al.* 2013). Another method using the localization information to estimate the resolution was reported as DAFL (Dai* et al.* 2016).

### Axial Localization and 3D imaging

The cellular structures are in 3D, but SMLM based on centroid fitting could only acquire 2D images of the sample, which will not satisfy the demand of biological research. So how to extend SMLM to the axial direction for imaging 3D cellular structures is one of the research hotspots in super-resolution techniques.

**Figure 2:**
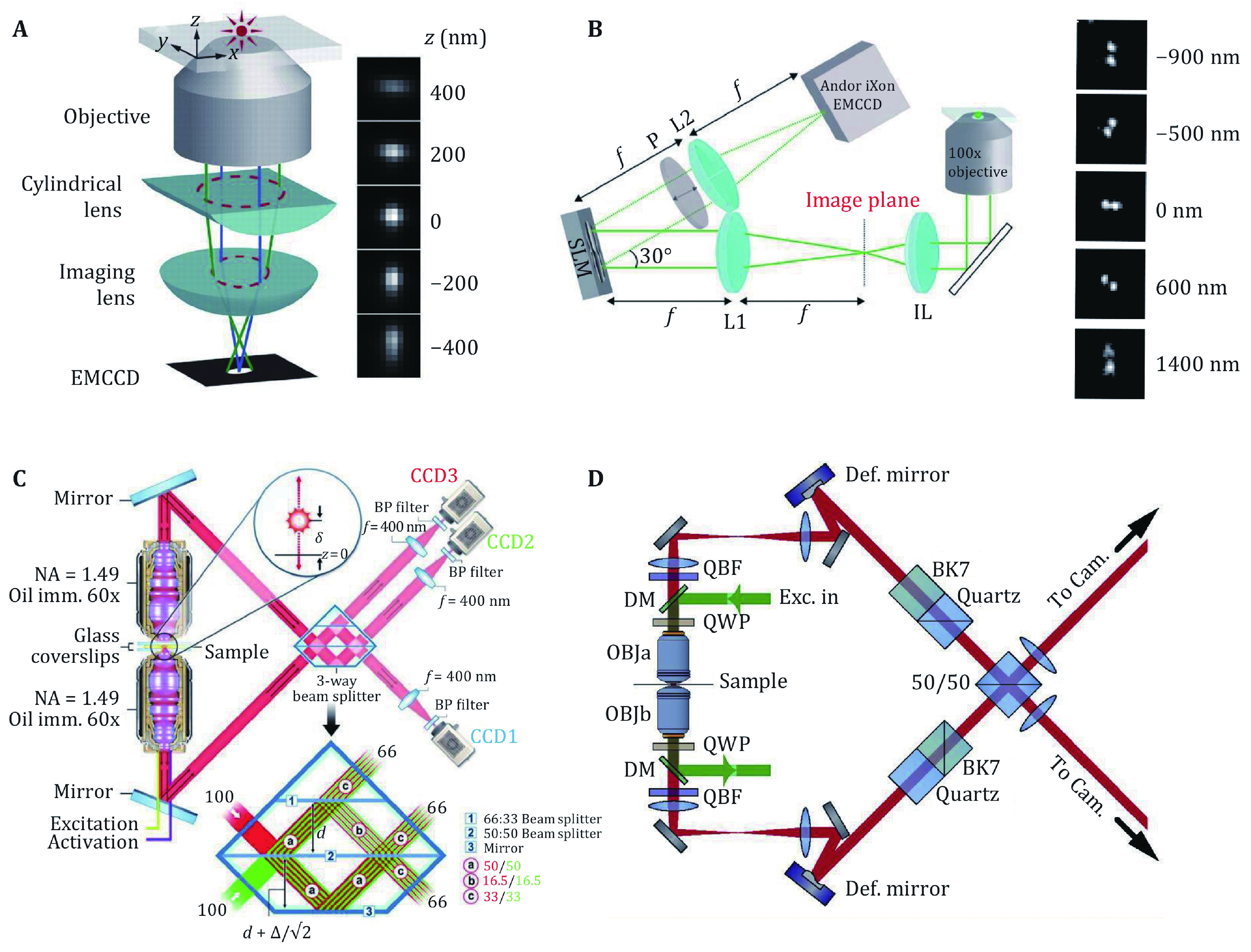
**A** Optical setup and the corresponding PSF of astigmatism based axial localization. Adapted from Zhu *et al*. (2012) with permission. **B** Optical setup and the corresponding PSF of the double-helix PSF for axial localization. Adapted from Badieirostami *et al*. (2010) with permission. **C** Optical setup and the interference prism of the iPALM. Adapted from Gustavsson *et al*. (2018) with permission. **D** Optical setup of 4Pi-SMS. Adapted from Shtengel *et al*. (2009) with permission

In 2008, Xiaowei Zhuang group reported the 3D STORM approach, in which a cylindrical lens was inserted in the imaging system to introduce astigmatism and change the shape of PSF with various focus depths (Huang* et al.* 2008a, b). The axial position of single molecule could be estimated by the shape of the PSF and the calibration curve (Fig. 2A). By introducing this method, the 3D shape of the target structure could be reconstructed. The lateral localization precision is about 10 nm and the axial localization precision is about 22 nm with this technique (Olivier* et al.* 2013; Xu* et al.* 2012).

Another 3D localization method was proposed as bi-plane (Badieirostami* et al.* 2010; Juette* et al.* 2008; Watanabe* et al.* 2011). In this method, the CCD was split into two parts, and two images of the same sample with the focus difference of about 500 nm were acquired. Thus, for each molecule, two images were acquired, and the 3D location could be calculated by the shape of the two images. Bi-plane yields a higher depth of field and more uniform localization precision across the depth of field, but the available area of CCD was halved (Min* et al.* 2014; Song* et al.* 2019; Winterflood* et al.* 2015).

Several 3D single-molecule localization approaches were based on the method of PSF engineering. The first demonstration of PSF engineering was double-helix PSF (Pavani and Piestun 2009; Pavani* et al.* 2009), which yields a two-point like PSF and the angle of the two points will change in different depths (Fig. 2B). This method shows the depth of field up to 2 µm, which is higher than astigmatism and the bi-plane approach (Backlund* et al.* 2013; Chen* et al.* 2013; Grover* et al.* 2011, 2012; Thompson* et al.* 2010). Recently, another PSF engineering method called Tetrapod was reported and the depth of field could be extended to several micrometers without large deterioration of localization precision (Gustavsson* et al.* 2018; Shechtman* et al.* 2015, 2017).

Imaging depth is an important aspect that is worth consideration. Inspired by the large propagation distance of airy beams, self-bending PSF was reported for a large depth of field in 3D single-molecule localization (Jia* et al.* 2014; Schroeder and Jia 2015; Zhou* et al.* 2020b). A side lobe free SB-PSF was designed, and the fluorescent signal was split into two parts, resulting in unambiguous 3D localization of fluorescent molecules. Imaging depth up to 3 µm was demonstrated and the 10–15 nm localization precision was achieved with a ~2000 photon budget.

When a molecule was located near the coverglass, the fluorescent will be enhanced by the additional signal within the super-critical angle. The axial localization could be determined by the ratio of super-critical signal and normal signal (Bourg* et al.* 2015; Deschamps* et al.* 2014). This method yields high axial localization precision near the coverslip surface, and the absolute localization is another unique characteristic of this method.

The relatively low axial localization precision mainly due to the collection angle range of the objective is limited in the half-con, resulting in less axial information compared with lateral directions. So, if imaging the sample with two opposite objectives, the additional axial information will increase the axial resolution. The first demonstration of this idea was so called interference PALM (iPALM), which uses a 3-way beam splitter to create three images of the sample with different phases (Fig. 2C) (Shtengel* et al.* 2009, 2014). With the high axial precision based on interferometric design and the doubled photon budget, both the lateral and axial resolutions were improved. This method was used to resolve the structure of cell adhesion (Bertocchi* et al.* 2017; Kanchanawong* et al.* 2010; Liu* et al.* 2015).

Another implementation was based on a 4Pi microscope, called 4Pi single-molecule switching (4Pi-SMS) (Aquino* et al.* 2011). In this method, a beam splitter was used to create interference and a polarized beam splitter was used to separate the p- and s-part of the fluorescence. Four images are generated and the axial localization of each molecule was determined by the intensity of the four images. When combined with adaptive optics to compensate for the aberrations induced by the sample, whole cell imaging with a high 3D resolution was achieved (Huang* et al.* 2016; Wang* et al.* 2021). It should be noticed that for the periodicity nature of interference. Thus, the additional axial localization technique was involved in the 4Pi-SMS method to resolve the periodicity of the interference.

One extraordinary work was reported as a combination of 4Pi-SMS and adaptive optics, which was named whole-cell 4Pi single-molecule switching nanoscopy (W-4PiSMSN) (Fig. 2D) (Huang* et al.* 2016). A high imaging depth with two color imaging ability was reported, which extends the imaging capabilities of 4Pi-SMS to whole cells without compromising resolution.

Another work on the 4Pi-SMS was focused on the multi-color imaging ability, which used a spectrum demixing strategy to recognize different types of the molecule (Zhang* et al.* 2020). With this method, 3-color imaging was achieved with negligible chromatic aberration.

The comparison of axial localization methods could be found in Table 1.

**Table 1:** Comparison of axial localization methods

Method	Working principle	Localization precision	Depth of field	Reference
Astigmatism	Introduce astigmatism to produce elliptical PSF	22 nm	~600 nm	Huang *et al*. 2008a
Bi-plane	Split image into two sub-images with different focus	~30 nm (resolution 75 nm)	1 μm	Juette *et al*. 2008
Double helix PSF	Use special light modulator to create double-helix PSF	10–20 nm	2 μm	Pavani *et al*. 2009
SB-PSF	Use special light modulator to create SB-PSF based on Airy beam	10–15 nm	3 μm	Jia *et al*. 2014
Super-critical angle fluorescent	Ratio-metric detection between normal signal and super-critical fluorescent signal	20 nm	150 nm	Bourg *et al*. 2015
iPALM	Fluorescent interference with two objectives	4.1 nm	300 nm	Shtengel *et al*. 2009
4Pi-SMS	Fluorescent interference with two objectives	2.3–3.5 nm	650 nm	Aquino *et al*. 2011
ROSE-Z	Laser based interferometric localization	2.4 nm	1 μm	Gu *et al*. 2021
ModLoc	Laser based interferometric localization	7.5 nm	1 μm	Jouchet *et al*. 2021

## SMLM WITH MINIMUM FLUXES

**Figure 3:**
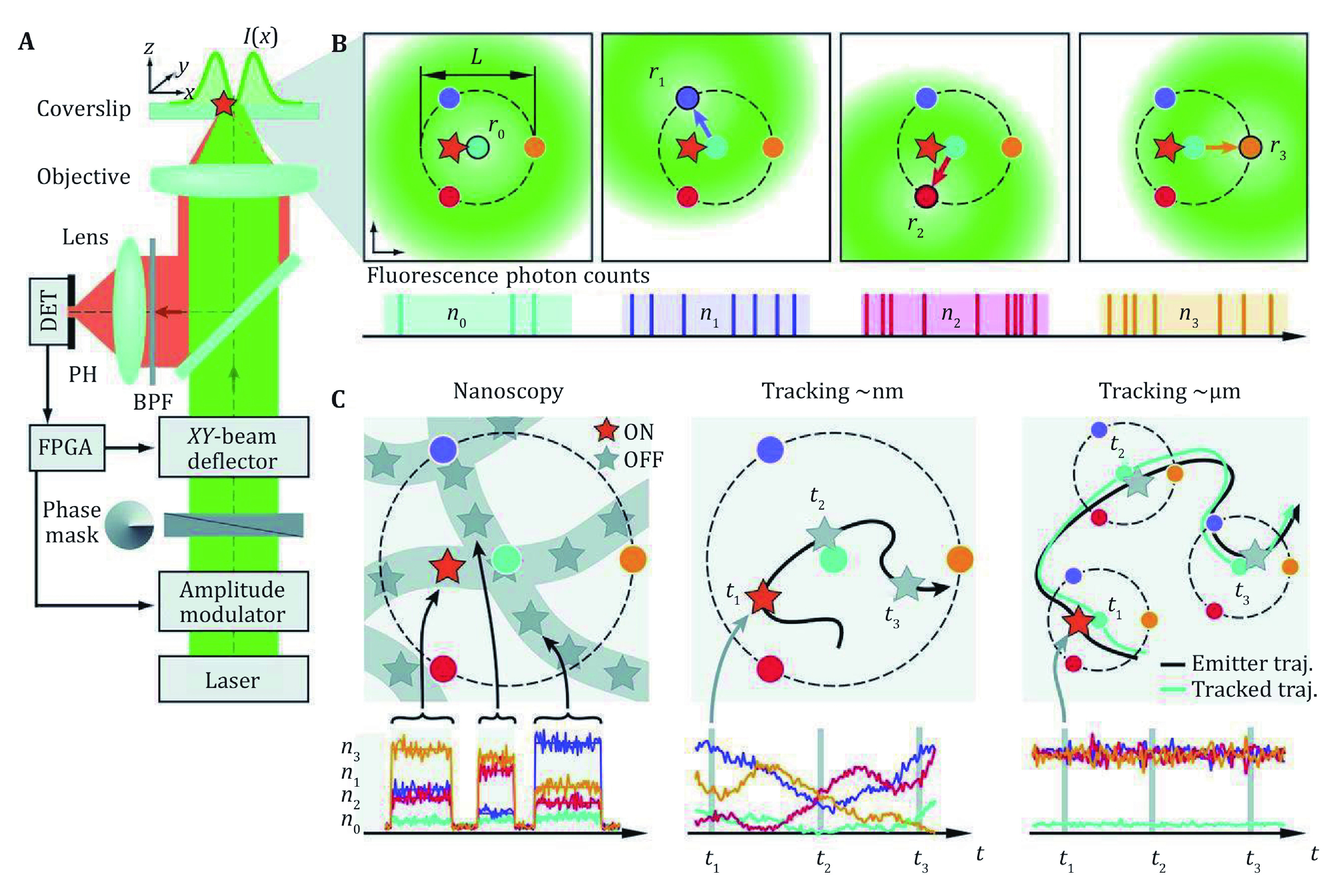
**A** Optical setup of MINFLUX for 2D detection. **B** Localization procedure of MINFLUX, the doughnut shaped spot was moved to four positions to measure the fluorescent signal. **C** MINFLUX could be applied in nanoscopy, single molecule tracking in nm range or a larger range. **A**, **B** and **C** are adapted from Liu *et al*. (2015) with permission

In 2017, a novel single-molecule localization method called MINFLUX was reported (Balzarotti* et al.* 2017; Sahl and Hell 2019). In this method, the single-molecule was excited by a doughnut shaped beam as used in STED, and the photon emitted from the molecule was used as the error signal rather than position signal as in conventional SMLM (Fig. 3A and 3B). This method could track single molecules with minimal photon fluxes, and the localization precision depends on the movement of the doughnut shaped beam (Eilers* et al.* 2018; Rocha* et al.* 2019). So as a consequence, the localization precision is not restricted by the width of PSF, and the resolving power was improved by an order.

The fast and accurate localization ability of MINFLUX makes it suitable for nanostructure resolving applications. The 1–2 nm localization precision could be achieved with only 1000 photons, and nanostructures of ~5 nm could be clearly resolved. On the other hand, the photon counting scheme makes it also suitable for high-speed single-molecule tracking. The localization speed up to 8 kHz was demonstrated with the tracking of single 30S ribosomal protein subunits fused to the switchable fluorescent protein mEos2 in *E. coli* bacteria, showing a localization precision around 50 nm with only nine photons per localization in mean. The superior spatiotemporal resolution provided by MINFLUX was also utilized to monitor the rapid molecular jumps with the measurement time as short as 400 microseconds and ~2 nm precision (Pape* et al.* 2020; Schmidt* et al.* 2021).

MINFLUX method was extended into the 3D imaging, which combined with a top hat shaped beam and iterative localization strategy. Together with the spectrum demixing method, multicolor imaging is also available, which successfully resolved the nuclear pore complex component Nup-96 and WGA simultaneously (Gwosch* et al.* 2020).

There's also a new method called p-MINFLUX, which used interleaved laser pulsed to deliver the doughnut-shaped excitation, yields a simplified experimental setup compared with MINFLUX, and additional fluorescence lifetime information could be extracted (Masullo* et al.* 2021).

## SMLM WITH STRUCTURED ILLUMINATION

Conventional single-molecule imaging utilizes either wide-field or TIR illumination, which results in a near uniformly distributed intensity profile for the excitation illumination. However, if additional information was added in the illumination, *i*.*e*. using a structured illumination rather than conventional uniform illumination, the information carried by the collected photons could be increased, and the localization precision could be improved compared with conventional single-molecule localization methods. As it’s hard to increase the photon budget further in SMLM, increasing the efficiency of the localization opens a new way for single-molecule localization.

It should be noticed that when utilizing structured illumination to locate single molecules, multiple measurements to the same molecule are required (six images were required for *X*–*Y* localization), and the localization was determined by the intensity rather than the shape of each molecule. If the molecule turned into a dark state or bleached during exposure, the acquired intensity will be affected, yielding higher location error.

**Figure 4:**
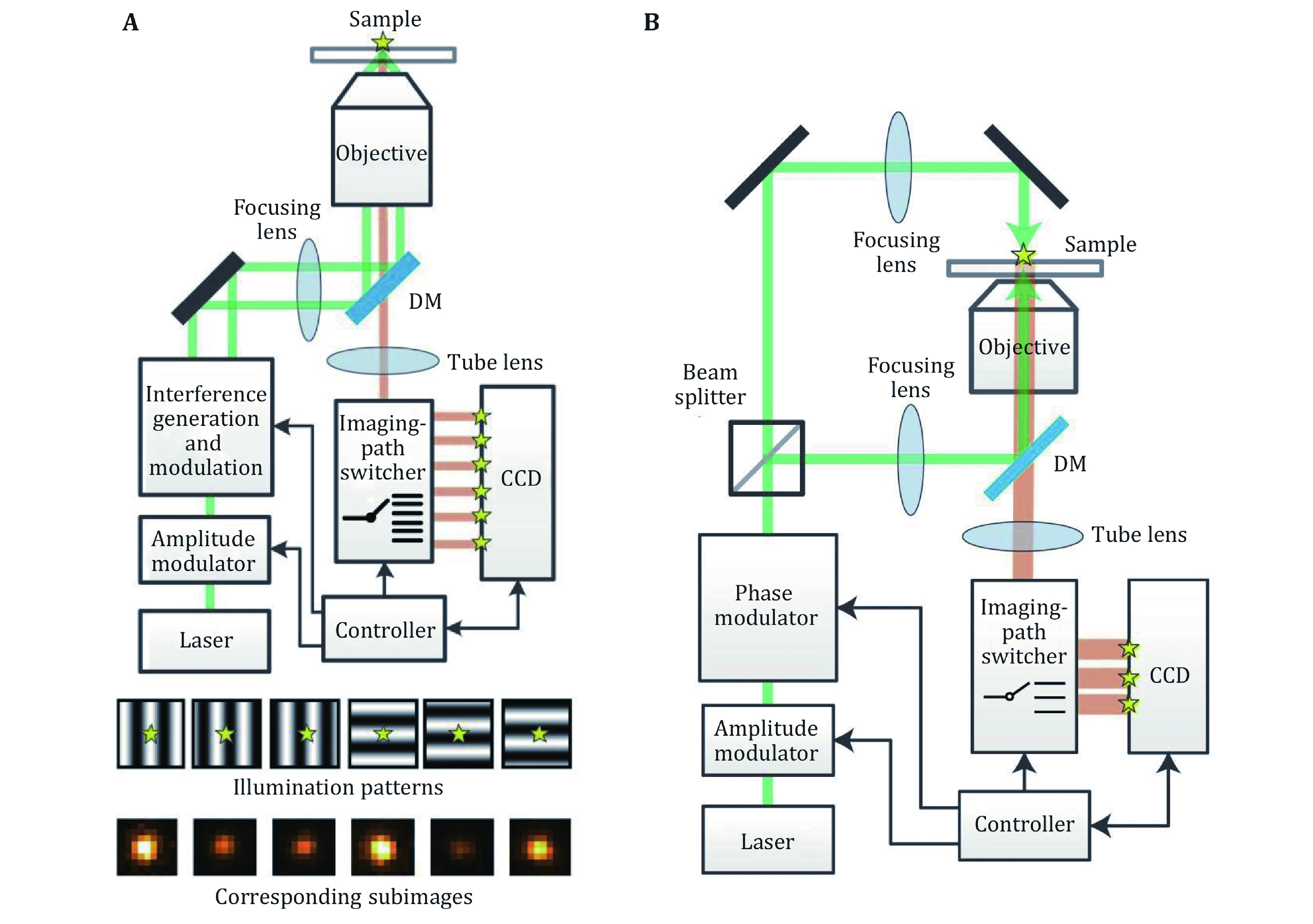
**A** Optical setup and the demonstration of six illumination patterns and six subimages for one single molecule in ROSE. Adapted from Eilers *et al*. (2018) with permission. **B** Optical setup of ROSE-Z, with asymmetric optics to create the axial interference pattern. Adapted from Rocha *et al*. (2019) with permission

One solution to realize the structured illumination localization idea and solve the difficulty of multi acquisition is a method reported as Repetitive Optical Selective Exposure (ROSE) (Gu* et al.* 2019). In this method, the imaging light path was specially designed such that the fluorescent signal was deflected by a resonant scanning mirror and focused on different areas of the CCD chip (Fig. 4A). The scanning mirror resonant at a frequency of about 4 kHz, resulting in an 8 kHz switching frequency, which is fast enough to eliminate the effect on localization precision due to the dynamic of single molecules. DNA-PAINT, as well as STORM imaging, was performed by ROSE, showing that ROSE could eliminate the effect of blinking and bleach during multi acquisition. With the structured illumination, the localization precision improved 2 to 2.4 times compared with the conventional localization method under the same photon budget, the resolution of SMLM could be enhanced.

The ROSE method was originally applied for the localization in *X*–*Y* directions. Recently there was a report of applying ROSE to the axial localization, which is called ROSE-Z (Gu* et al.* 2021). In this method, an asymmetric optical path was used to generate an axially distributed interference pattern for the localization, and a 3-way ROSE detection optical path was used for imaging (Fig. 4B). With this method, the axial localization precision was improved 6–8 times compared with the conventional astigmatism method, which is even higher than the lateral localization. This kind of axial localization precision was only achievable by iPALM or 4Pi-SMS with two objectives before. Furthermore, when combined with spectrum demixing, two color imaging was also demonstrated without the effect of chromatic aberration.

Another reported method named structured illumination microscopy based point localization estimator (SIMPLE) was a straightforward implementation of the structured illumination localization (Reymond* et al.* 2019). In this work, a digital micro-mirror device (DMD) was used as a spatial light modulator, to create precisely phase-shifted sinusoidal wave patterns as nanometric rulers, and molecules were localized by the intensity under each phase. In this method, the six acquisitions were performed sequentially, meaning that it’s not appliable for STORM imaging of highly dynamic molecules.

There’s another similar method named SIMFLUX, which used a pair of gratings to generate the sinusoidal illumination pattern along *X*- and *Y*-directions, and for each grating, a PIEZO was used for the phase shifting (Cnossen* et al.* 2020). A Pockels cell was used to switch the laser beam between the two light paths, and the PIEZO in the inactive light path could have enough time to move to the next phase. This design enables cycling through six patterns (two orientations, three phase steps) on the millisecond time scale with sufficient power throughput. With the acquisition taken sequentially as SIMPLE, SIMFLUX still suffers from the dynamics of molecules and yields some limitations in the SMLM imaging.

A 2-fold improvement of localization precision was reported by several works, compared with the conventional centroid estimation methods. SIMPLE was validated by immobilized Alexa Fluor 488 molecules, and the result showed a 2–2.4 fold improvement. SIMFLUX was validated with DNA-PAINT nano-rulers and STORM imaging. Compared with MINFLUX, the parallelized localization scheme increased the throughput dramatically, but the improvement of localization precision was limited to about 2-fold.

There’s another fluorescent acquisition method published as Modulated Localization microscopy (ModLoc) (Jouchet* et al.* 2021). This method introduced a Pockels cell to change the polarization status of the fluorescent signals, then a polarization beam splitter was used to switch the fluorescent signal to the different parts of the CCD chip. The modulation/demodulation frequency was typically 600 Hz, which is designed to be compatible with the short ON-time of the emitters. The ModLoc generates four subimages, which were used for the axial localization of single molecules and shown a high axial resolution even at the depth of several microns.

A summarized comparison of the methods in this section could be found in Table 2.

**Table 2:** Comparison of SMLM methods with structured illumination

Method	Working principle	Switching time per cycle	Localization precision	Reference
ROSE/ROSE-Z	Ultra-fast switching between subimages with resonant scanner mirror	125 μs	*XY*: 2–2.4 nm (ROSE)*Z*: 2.4 nm (ROSE-Z)	Gu *et al*. 2019, 2021
SIMPLE	Sychronized DMD and sCMOS to image single molecule several times with different illuminatin patterns	300 ms (50 ms exposure time)	*XY*: 4.7 nm	Reymond *et al*. 2019
SIMFLUX	Ping Pong operation is carried out between the two optical paths to avoid the setting time of PIEZO	30–400 ms	*XY*: 7.4–9.6 nm	Cnossen *et al*. 2020
ModLoc	The fluorescent beam was modulated by a Pockels cell, then switched by polarization beam splitter	10 ms	*XY*: 3.3 nm*Z*: 7.5 nm	Jouchet *et al*. 2021

## SUMMARY AND PERSPECTIVES

With characteristics of high spatial resolution, high specificity and non-invasive, SMLM has been widely used in life sciences. In this review, we attempt to introduce recent developments of SMLM from technical aspects. With this powerful tool, researchers could resolve nanostructures once hidden behind the diffraction limit of light microscopy. Besides the great achievements of super-resolution methods, there’s still a lot of room for improvement. With the development in optical design, better detector, new dyes and analysis algorithms, this method will become more and more powerful and will extend the application of SMLM into biomacromolecule structure study.

## Conflict of interest

Lusheng Gu and Wei Ji declare that they have no conflict of interest.
